# KRAB–Zinc Finger Proteins and KAP1 Can Mediate Long-Range Transcriptional Repression through Heterochromatin Spreading

**DOI:** 10.1371/journal.pgen.1000869

**Published:** 2010-03-05

**Authors:** Anna C. Groner, Sylvain Meylan, Angela Ciuffi, Nadine Zangger, Giovanna Ambrosini, Nicolas Dénervaud, Philipp Bucher, Didier Trono

**Affiliations:** 1 School of Life Sciences, École Polytechnique Fédérale de Lausanne (EPFL), Lausanne, Switzerland; 2 Frontiers-in-Genetics National Center of Competence in Research, École Polytechnique Fédérale de Lausanne (EPFL), Lausanne, Switzerland; 3 Institute of Microbiology, University Hospital Center and University of Lausanne, Lausanne, Switzerland; 4 Swiss Institute of Bioinformatics, Lausanne, Switzerland; University of California San Francisco, United States of America

## Abstract

Krüppel-associated box domain-zinc finger proteins (KRAB–ZFPs) are tetrapod-specific transcriptional repressors encoded in the hundreds by the human genome. In order to explore their as yet ill-defined impact on gene expression, we developed an ectopic repressor assay, allowing the study of KRAB–mediated transcriptional regulation at hundreds of different transcriptional units. By targeting a drug-controllable KRAB–containing repressor to gene-trapping lentiviral vectors, we demonstrate that KRAB and its corepressor KAP1 can silence promoters located several tens of kilobases (kb) away from their DNA binding sites, with an efficiency which is generally higher for promoters located within 15 kb or less. Silenced promoters exhibit a loss of histone H3-acetylation, an increase in H3 lysine 9 trimethylation (H3K9me3), and a drop in RNA Pol II recruitment, consistent with a block of transcriptional initiation following the establishment of silencing marks. Furthermore, we reveal that KRAB–mediated repression is established by the long-range spreading of H3K9me3 and heterochromatin protein 1 β (HP1β) between the repressor binding site and the promoter. We confirm the biological relevance of this phenomenon by documenting KAP1–dependent transcriptional repression at an endogenous KRAB–ZFP gene cluster, where KAP1 binds to the 3′ end of genes and mediates propagation of H3K9me3 and HP1β towards their 5′ end. Together, our data support a model in which KRAB/KAP1 recruitment induces long-range repression through the spread of heterochromatin. This finding not only suggests auto-regulatory mechanisms in the control of KRAB–ZFP gene clusters, but also provides important cues for interpreting future genome-wide DNA binding data of KRAB–ZFPs and KAP1.

## Author Summary

The regulation of gene activity by transcription factors is crucial to the function of all cells. Here, we studied the mechanisms of action of the largest family of gene regulators encoded by the human genome, the so-called KRAB–containing zinc finger proteins (KRAB–ZFPs), which in concert with their universal cofactor KAP1 act as transcriptional repressors. For this, we used two parallel approaches. First, by targeting an ectopic KRAB domain to hundreds of different genes, we found that KRAB/KAP1 can repress promoters located several tens of kilobases from the repressor DNA docking site. We further could show that KRAB induces such long-range effects by mediating the spread of repressive chromatin marks along the body of the gene, resulting in a block of transcriptional initiation at the promoter. In a second set of experiments, we analyzed an endogenous KRAB–ZFP gene cluster, where we could also document KAP1–dependent heterochromatin spreading and transcriptional repression. Together, these results support a model whereby KRAB–ZFPs and KAP1 can mediate long-range transcriptional repression through the spread of silencing chromatin marks. This study thus provides insight into KRAB/KAP1–induced gene regulation at KRAB–ZFP gene clusters, and will further help interpret genome-wide studies of KRAB–ZFPs and KAP1 DNA binding patterns.

## Introduction

The proper control of gene expression is paramount to all cellular events, and is orchestrated through a sophisticated balance of activating and repressing influences. Krüppel-associated box domain zinc finger proteins (KRAB-ZFP) constitute the single largest group of transcriptional repressors encoded by the genomes of higher organisms. After appearing in early tetrapods, the KRAB-ZFP family has rapidly expanded and diverged through multiple rounds of gene and segmental duplications, to give rise to more than three hundred and fifty members annotated in both mice and humans [1]–[6]. In spite of their numerical abundance, wide range of tissue-specific expression and dynamic evolutionary history, the physiological functions of KRAB-ZFPs collectively remain ill-defined, and few of their targets have been identified [3],[7]. However, emerging evidence links KRAB/KAP1-mediated regulation to processes as essential and diverse as stem cell pluripotency, early embryonic development and differentiation, genomic imprinting, response to DNA damage and control of behavioral stress [8]–[14]. Furthermore, KAP1 controls endogenous retroviruses in embryonic stem cells, a process crucial for the maintenance of genomic stability [15].

KRAB-ZFPs all harbor a so-called KRAB domain situated upstream of an array of two to forty C2H2 zinc fingers, which provide sequence-specific DNA binding ability [2]. KRAB recruits KAP1 (KRAB-associated protein 1, also known as TRIM28, Tif1β or KRIP-1) [16]–[19], which acts as a scaffold for various heterochromatin-inducing factors, such as heterochromatin protein 1 (HP1), the histone methyltransferase SETDB1, the nucleosome-remodeling and histone deacetylation (NuRD) complex, the nuclear receptor corepressor complex 1 (N-CoR1) and, at least during early embryonic development, *de novo* DNA methyltransferases [20]–[27].

The phylogenetically conserved family of HP1 proteins is implicated in a variety of nuclear events, such as transcriptional repression and maintenance of chromosome structure [28]. HP1 harbors two major regions: the chromo domain, which binds to repressive di- and trimethylated histone 3 lysine 9 (H3K9me2 and H3K9me3, respectively) residues, and the chromo shadow domain, involved in HP1 homodimerization and recruitment of other partners [29]–[31]. Therefore, HP1 bridges histones with other chromatin-associated proteins, that promote heterochromatin spreading [32]. An advancing front of heterochromatization can thus be propagated by the creation of HP1 binding sites, through histone methyltransferase-mediated H3K9 methylation, followed by the HP1-mediated recruitment of more histone methyltransferase for another round of H3K9 methylation/HP1 binding [33]–[36]. Little is known about the kinetics, efficiency, self-perpetuating ability and action-range of this process. Tethering of a regulated KRAB repressor domain suggested that KRAB/KAP1 induced heterochromatin formation has a rather limited spreading potential in euchromatin, as silencing could be exerted no farther than 2–3 kilobases (kb) away from the repressor binding site [37]. Yet a more recent Chromatin IP (ChIP)-on-chip analysis performed in the human testicular carcinoma cell line Ntera2 revealed close to 7,000 KAP1 binding sites, a number of which were located at the 3′end of KRAB-ZFP genes [38]. While this suggests auto-regulatory negative feedback loops for these genes, such a process would imply that KRAB/KAP1 binding can affect promoters situated at very significant distances. In agreement with such a model, large heterochromatin domains associated with both HP1β and SUV39h1 were found on chromosome 19, where most of the KRAB-ZFP gene clusters reside [39].

To examine the genomic features of KRAB/KAP1-mediated transcriptional regulation, we developed an ectopic repressor assay. In our system, promoterless lentiviral vectors serve as gene traps to drive reporter expression from cellular promoters. Drug-controllable docking of an ectopic KRAB-based repressor then allows an assessment of the effects of KRAB/KAP1 recruitment at these loci. Using this system, we found that KRAB-induced silencing can repress promoters situated several tens of kilobases away from the repressor primary docking site through reduced RNA Pol II binding. Furthermore, we observed that this phenomenon is independent of promoter strength, facilitated by HP1 and associated with spreading of heterochromatin marks between repressor binding site and targeted promoters. Finally, we could document KAP1-mediated transcriptional repression at an endogenous KRAB-ZFP gene cluster by propagation of HP1β and H3K9me3 from the 3′ end of these genes to their transcriptional start site. Our results indicate that KRAB/KAP1 induce long-range repression through the spread of heterochromatin.

## Results

### KRAB–mediated silencing can act over several tens of kilobases

**Figure 1:**
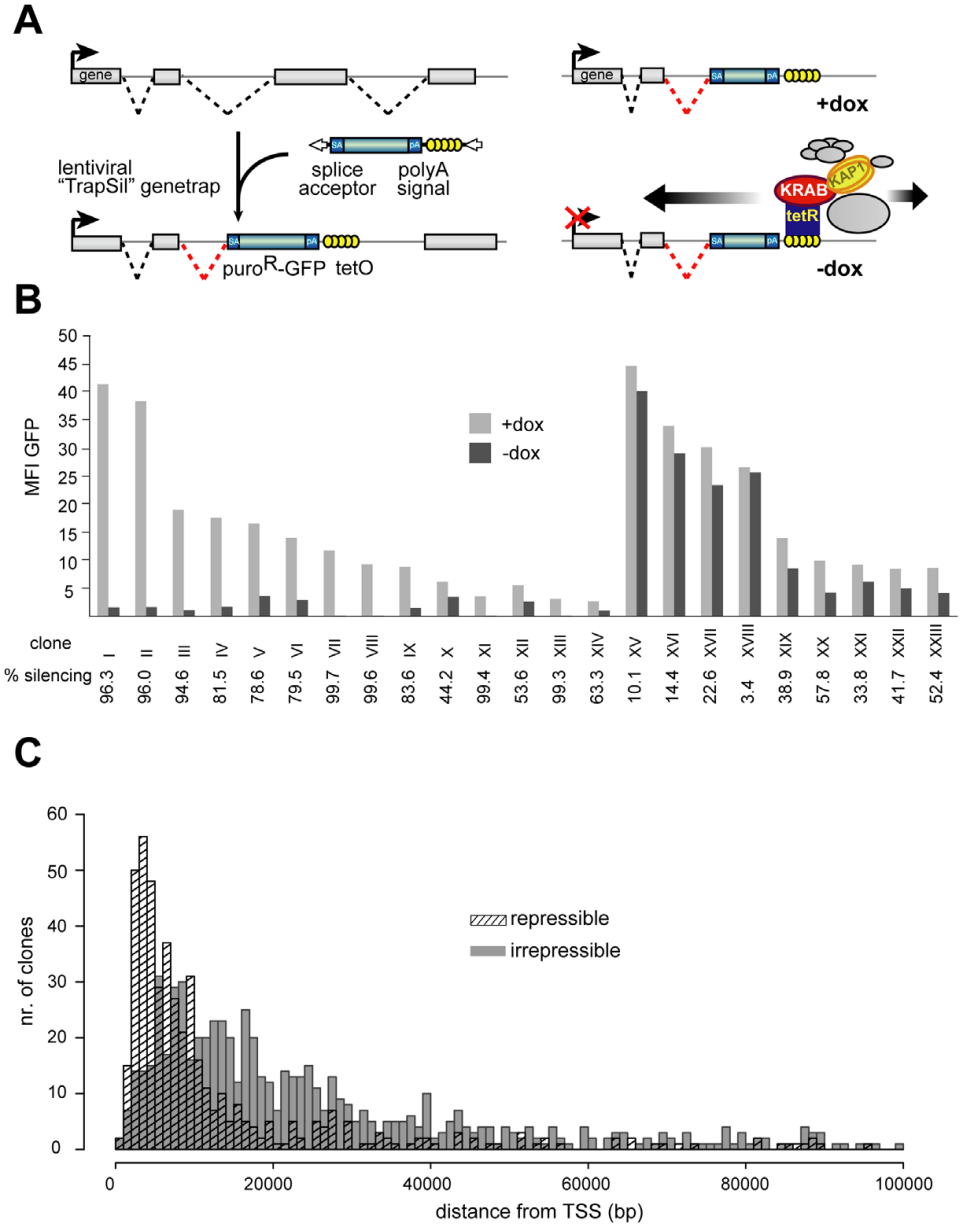
KRAB–mediated silencing can act over several tens of kilobases. (A) Mechanism of how endogenous genes are targeted by tTRKRAB using the lentiviral vector-based “Trapping/Silencing” (TrapSil) system: *TetO*-containing gene traps carrying the promoterless puro^R^-GFP gene only express this reporter if after proviral integration they “trap” an actively transcribing gene. The *TetO* sites further allow binding of the ectopic repressor tTRKRAB to the gene traps after dox removal, while the trap reporter serves as a direct read-out for the effects of tTRKRAB–mediated “silencing”. (B) tTRKRAB–expressing HeLa cells were transduced with LV TrapSil vectors and 23 clones expressing the trap reporter were isolated. Mean fluorescence intensity (MFI) GFP values of these individual clones, cultured with and without dox, were determined and the ratios of these values were used to calculate the silencing efficiency (% silencing  = 1- ((MFI GFP –dox)/(MFI GFP +dox))) depicted below the x-axis for each clone. (C) GFP-mediated cell sorting was used to isolate populations of HeLa cells exhibiting either a “repressible” (>90% silencing) or an “irrepressible” (<10% silencing) phenotype. 484 repressible and 699 irrepressible clonal integrations were mapped and the distance between them and their trapped promoter was plotted on a cumulative histogram.

In order to study the features of KRAB/KAP1-induced silencing, we exposed lentivirally trapped cellular promoters to a drug-regulated KRAB-containing repressor. The tTRKRAB protein contains the KRAB domain of the human KOX1 ZFP fused to the *E. coli* tetracycline repressor (tTR), and binds to *Tet* operator sequences (*TetO*) in a doxycycline (dox)-controllable fashion [40],[41]. We engineered human immunodeficiency virus (HIV)-derived lentiviral (LV) gene trap vectors carrying tandem *TetO* repeats and a promoter-less puromycin resistance-GFP fusion reporter (puro^R^-GFP) downstream of a potent adenoviral splice acceptor. This design predicted that i) reporter expression would occur from the promoters of active genes targeted by the integrants, and ii) dox withdrawal would result in tTRKRAB binding to the *TetO* sites present in the proviruses, thus exposing the trapped promoters to potential KRAB/KAP1-mediated repression (Figure 1A).

To study the effects of tTRKRAB recruitment to endogenous genes, we engineered HeLa cells stably expressing tTRKRAB and infected those with the *TetO*-containing LV trapping/silencing (“TrapSil”) vectors at a low multiplicity of infection to limit integrants to one per cell. Clones expressing the trap reporter puro^R^-GFP were then isolated and subjected to FACS analysis in the presence (-dox) or absence (+dox) of tTRKRAB binding. Comparing the mean fluorescence intensity values of GFP (MFI) in both conditions revealed heterogeneous effects of tTRKRAB recruitment, with clones exhibiting either high or low levels of reporter silencing (Figure 1B and Table S1). We saw no correlation between the strength of the trapped promoters and their susceptibility to tTRKRAB-mediated repression (Figure 1B). We could also verify proper recruitment of KRAB/KAP1 in different clones by ChIP targeting KAP1 (Figure S1 and data not shown). In addition, we analyzed the effects of tTRKRAB recruitment on populations of LV TrapSil clones and found that the level of GFP was decreased on both the protein and mRNA level upon repressor binding. We also found that the average silencing efficiency of the LV TrapSil population was at around 60%, which is consistent with repressible clones being more prevalent in this population than irrepressible clones (Figure S2).

HIV-derived vectors preferentially integrate in downstream regions of transcribed genes [42],[43]. Therefore, we evaluated the impact of distance between the tTRKRAB docking site and the trapped promoter on KRAB-induced repression in cell populations with distinct LV TrapSil integrants. For this, we used GFP-based cell sorting to purify subpopulations exhibiting trapped promoters, which were silenced by either more than 90% (“repressible”) or less than 10% (“irrepressible”). Proviral integration sites were mapped by ligation-mediated PCR on genomic DNA, followed by large scale pyrosequencing [44],[45]. To ensure an unequivocal analysis, we chose to consider only trapping events that mapped to characterized UCSC known genes with single transcriptional start sites. Using these criteria we were able to obtain 484 repressible integrants in 221 genes and 699 irrepressible integrants in 371 genes (Table S2). When we compared the distance between the proviral integration sites and their trapped promoters in both populations, we found that irrepressible promoters were on average located further away from the repressor-docking site than their repressible counterparts (Figure 1C). We observed a similar pattern when analyzing trapped genes harboring multiple integration events from both the repressible and the irrepressible groups. By analyzing six of the most targeted genes, we found that the provirus integrated closest to the promoter systematically induced a repressible phenotype, while integrants located further downstream often fell into the irrepressible group (Figure S3). The comparison between the effect of tTRKRAB-mediated silencing and the range of its action also revealed that repression could be exerted at several tens of kilobases away from the tTRKRAB binding site, albeit with a sharp decrease at around 15 kb (Figure 1C). Finally, the overlap between the two histograms indicates that distance between repressor-binding site and the targeted promoter was not the sole determinant of susceptibility to KRAB-mediated repression, but that sequence- or locus-dependent factors must also play a role.

### Silenced promoters exhibit increased H3K9me3 and decreased H3-acetylation and RNA Pol II recruitment

**Figure 2:**
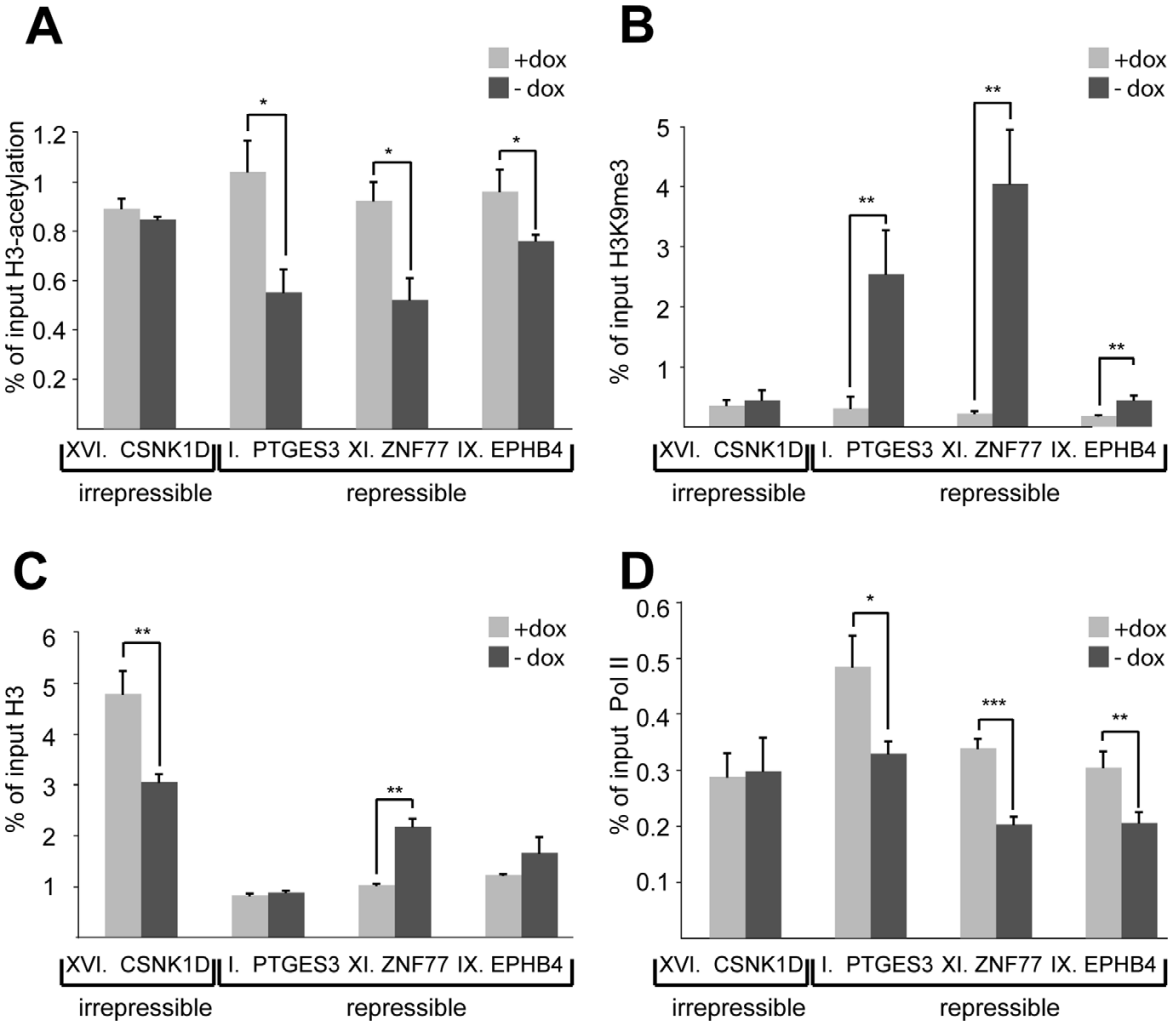
Silenced promoters exhibit increased H3K9me3 and decreased H3-acetylation and RNA Pol II recruitment. ChIP–based measurement of (A) H3-acetylation, (B) H3K9me3, (C) total histone H3, and (D) hypophosphorylated RNA Pol II at the irrepressible promoter of casein kinase 1d (CSNK1D) and at the repressible promoters of prostaglandin E synthase 3 (PTGES3), zinc finger protein 77 (ZNF77) and ephrin receptor B4 (EPHB4). The relative enrichment values (% of input) of H3-acetylation, total H3, and RNA Pol II were normalized to the relative enrichment at the GAPDH promoter. All values are expressed as means +SEM of triplicate experiments. The statistical test conducted was the student's t-test comparing +dox to –dox samples. If unequal variances were observed, the values were log10 transformed. The criterion for significance for all analyses was p<0.05. *: p<0.05, **, p<0.01, ***, p<0.005.

To investigate the molecular bases of repressible and irrepressible phenotypes we characterized four clones (I, IX, XI and XVI) in more detail using 5′RACE to map the trapped fusion transcripts (Table S1) and by performing ChIP studies. All clones had the integrant within 10–20 kb from the trapped promoter, yet three (clones I, IX and XI) were repressible and one (clone XVI) was irrepressible (Figure 1B). The level of histone modifications known to be affected by KRAB/KAP1-mediated repression, namely histone H3-acetylation (H3Ac) and H3K9me3 was evaluated at each of the four trapped promoters. In all three repressible clones, H3Ac levels dropped significantly upon tTRKRAB-binding, consistent with a close to complete deacetylation of the trapped allele (Figure 2A). In contrast, the irrepressible promoter of the casein kinase 1d (CSNK1D) gene, trapped in clone XVI, exhibited constant levels of this chromatin mark (Figure 2A). An inverse image was obtained for H3K9me3, with increases of different magnitudes at the three silenced promoters upon dox removal. No such change in H3K9me3 levels was observed at the irrepressible promoter (Figure 2B). To exclude the possibility that increased H3K9me3 was due to chromatin condensation, we also monitored levels of total histone H3 at these promoters. Comparison of the dox+ and dox- conditions indicated that the observed H3K9me3 increases could not be solely attributed to changes in total H3 (Figure 2C). In order to probe the impact of KAP1-induced repression on transcriptional initiation, we measured the levels of hypophosphorylated RNA Pol II present at trapped promoters (Figure 2D). We found that tTRKRAB binding resulted in a significant decrease of the recruitment of this enzyme in repressible but not irrepressible clones. Noteworthy, the remaining levels of Pol II binding detected in the absence of dox most likely originated from the sister allele of the trapped promoter.

### HP1β and H3K9me3 spread from the KRAB–binding site to the promoter

To investigate further the mechanisms of this long-range repression, we measured levels of both H3K9me3 and HP1β enrichment between the promoters and the TrapSil integrants of the loci under study. It was previously demonstrated that all HP1 isoforms can interact with KAP1 [20],[22],[29],[46],[47]. However, recent FRET results revealed distinct physical interactions between KAP1 and HP1β or HP1γ during differentiation of embryonal carcinoma F9 cells to primitive endoderm-like cells [48]. Interestingly, only the KAP1-HP1β interaction induced a relocalization of genes from euchromatin to heterochromatin upon differentiation, suggesting a specific role for this isoform during KAP1-mediated gene silencing. In addition, both HP1β and KAP1 co-localize at the 3′end of KRAB-ZFP genes [38],[39], making HP1β a likely candidate to be involved in propagating KRAB/KAP1-mediated long-range repression.

**Figure 3:**
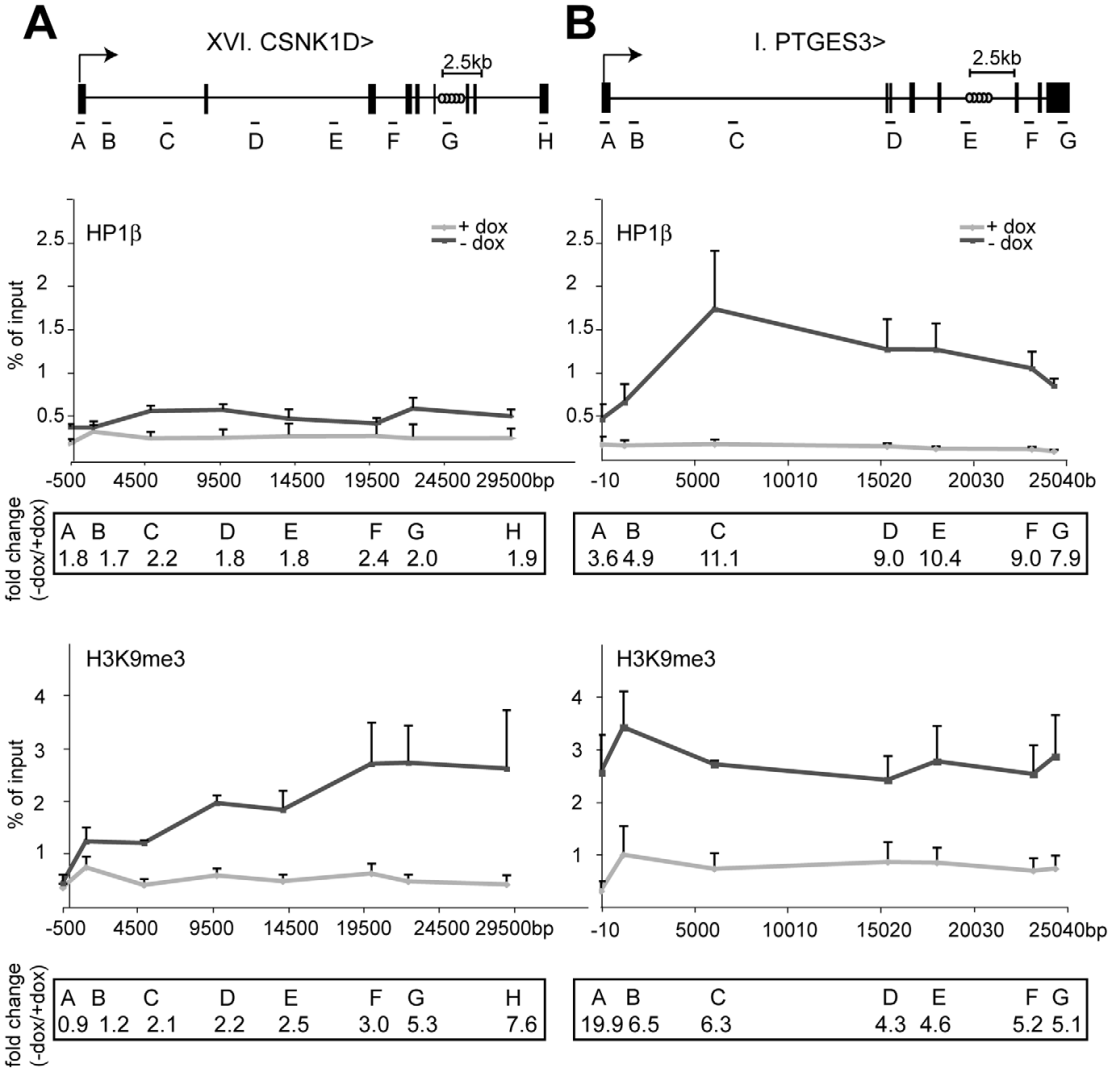
HP1β and H3K9me3 spread from the KRAB–binding site to the promoter. ChIP analyses quantifying the relative enrichment (% of input) of both H3K9me3 and HP1β were performed for the irrepressible clone XVI and the repressible clone I in the presence and absence of dox. The interrogated sequence spanned from the proviral tTRKRAB binding sites (light grey circles) to the indicated trapped promoters. (A) Enrichments of both modifications at the trapped casein kinase 1d (CSNK1D) locus. (B) Enrichments of both modifications at the prostaglandin E synthase 3 (PTGES3) locus. qPCR amplicons are depicted as letters and are not drawn to scale. All values are expressed as means +SEM of triplicate experiments. Fold changes were calculated as ratios of -dox/+dox enrichments, with the ratios of the respective positive controls set as 1. The controls consisted of p53BP2 for H3K9me3 and of ZNF556 for HP1β (Table S4).

Indeed, global levels of H3K9me3 and HP1β increased along the body of trapped genes upon tTRKRAB recruitment, and the magnitude and efficiency of propagation of this phenomenon correlated with its phenotypic outcome (Figure 3, Figure S4, Figure S5). Clone I, where the prostaglandin E synthase 3 (PTGES3) gene was trapped, exhibited high reporter GFP expression, which was completely silenced upon tTRKRAB binding (Figure 1B). Correspondingly, KRAB repressor binding led to a massive increase of both HP1β and H3K9me3 along this gene (Figure 3B). In comparison, the irrepressible CSNK1D gene body, showed smaller changes in HP1β and H3K9me3 loading, which gradually decreased towards the promoter (Figure 3A). The ChIP efficiencies between the +dox and the –dox samples were comparable for both factors, as shown by analysis of several control loci (Figure S6). We additionally monitored the levels of H3K9me3 and HP1β at the trapped and repressible KRAB-ZFP gene ZNF77. More specifically, tTRKRAB binding to the 3′end of ZNF77-1 led to an increase of both factors at the promoters and gene bodies of ZNF77-1, as well as the neighboring ZNF57 gene, which is located approximately 40 kb away (Figure S5). Therefore, we conclude that KRAB/KAP1 recruitment triggers the long-range spreading of silent chromatin marks. Efficient transcriptional silencing, however, is only established when this process extends over the promoter itself, interfering with proper RNA Pol II recruitment and transcriptional initiation (Figure 2D).

### HP1 facilitates KRAB/KAP1–mediated long-range repression

**Figure 4:**
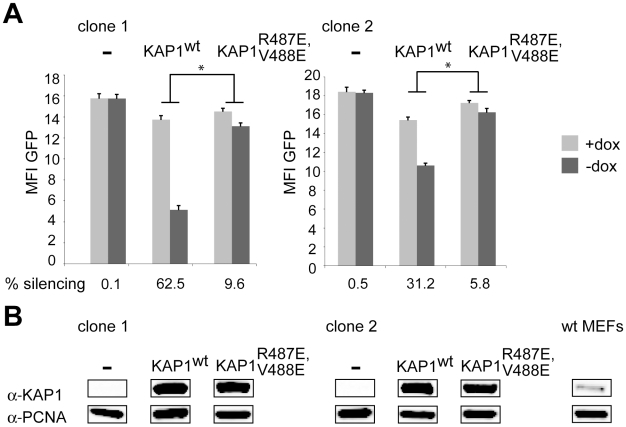
HP1 facilitates KRAB/KAP1–mediated long-range repression. (A) Two LV TrapSil-selected, tTRKRAB–expressing KAP1^−/−^ MEF clones were complemented by transduction with a lentiviral vector expressing either wild type KAP1 (KAP1^wt^) or HP1 binding-defective KAP1 (KAP1^R487E, V488E^), before FACS analysis in the presence or absence of dox. The mean fluorescence intensity values (MFI) of GFP and the silencing efficiency values are depicted as means of six independent measurements. The statistical analysis was performed using two-way ANOVA and, since the interactions were linked, the simple main effects were analyzed. The criterion for significance for all analyses was p<0.05. *: p<0.05. (B) Western blot analysis monitoring KAP1 protein levels, using PCNA as a loading control. The far right panel shows endogenous KAP1 expression in a MEF wild-type cell line.

We further explored the role of HP1 recruitment to KAP1 in KRAB/KAP1-mediated long-range repression by performing complementation studies in a KAP1 knockout mouse embryonic fibroblast (MEF) cell line stably expressing tTRKRAB. For this, we isolated two LV TrapSil clones, and used 5′RACE to map the fusion transcripts resulting from promoter trapping. Since both proviruses were intronic, we assigned integration to the middle of the targeted intron. This revealed that clone 1 had integrated in the gene Sestd1 (SEC14 and spectrin domains 1) at a distance of 15 kb from the promoter and that clone 2 had trapped the gene Prcp (prolylcarboxypeptidase) and was situated 25 kb away from its TSS (Table S1). Both clones were therefore suitable to serve in our study of KRAB/KAP1-mediated long-range repression. We found that GFP expression was independent of the presence of dox (Figure 4A), as predicted from the knockout of KAP1 (Figure 4B). However, silencing could be significantly restored in both clones by lentiviral vector-mediated complementation with wild type KAP1 (Figure 4A and 4B). By comparison, the transduction with a vector expressing a KAP1 mutant defective for HP1 binding (KAP1^R487E, V488E^) [22] only rescued tTRKRAB-mediated silencing to levels that were about six times lower than observed with wild type KAP1 in both clones (Figure 4A and 4B). This strongly suggests that HP1 recruitment by KAP1 facilitates the long-range impact of the KRAB/KAP1 silencing complex.

### KAP1 represses KRAB-ZFP genes via 3′ to 5′ long-range heterochromatin spreading

**Figure 5:**
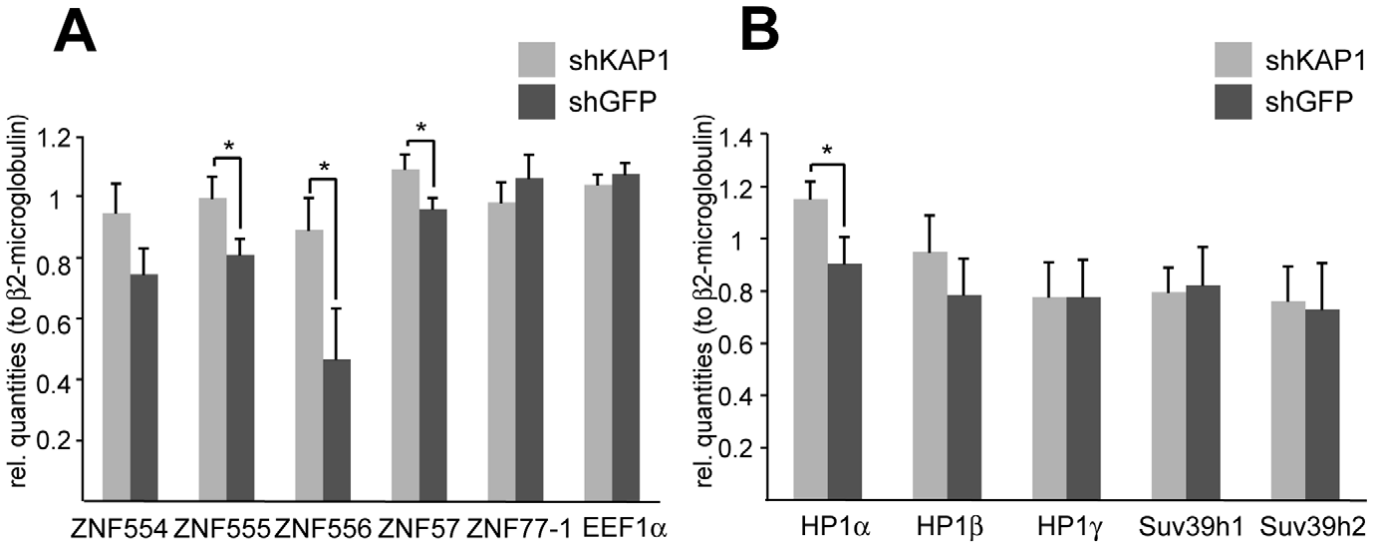
KAP1 mediates transcriptional repression of KRAB–ZFPs. mRNA expression levels upon stable KAP1 (shKAP1) and GFP (shGFP) knockdown were assessed through qPCR in HeLa cells. The relative quantities were measured for (A) cluster-specific KRAB–ZFP transcripts and for (B) different heterochromatin protein expression levels. β2-microglobulin was used as a normalization gene, whereas the expression of the eukaryotic elongation factor 1α (EEF1α) served as a negative control. qPCR values are expressed as means +SEM of six experiments. The statistical test conducted was a t-test with Welch's correction, which assumes unequal variances: *: p<0.05.

**Figure 6:**
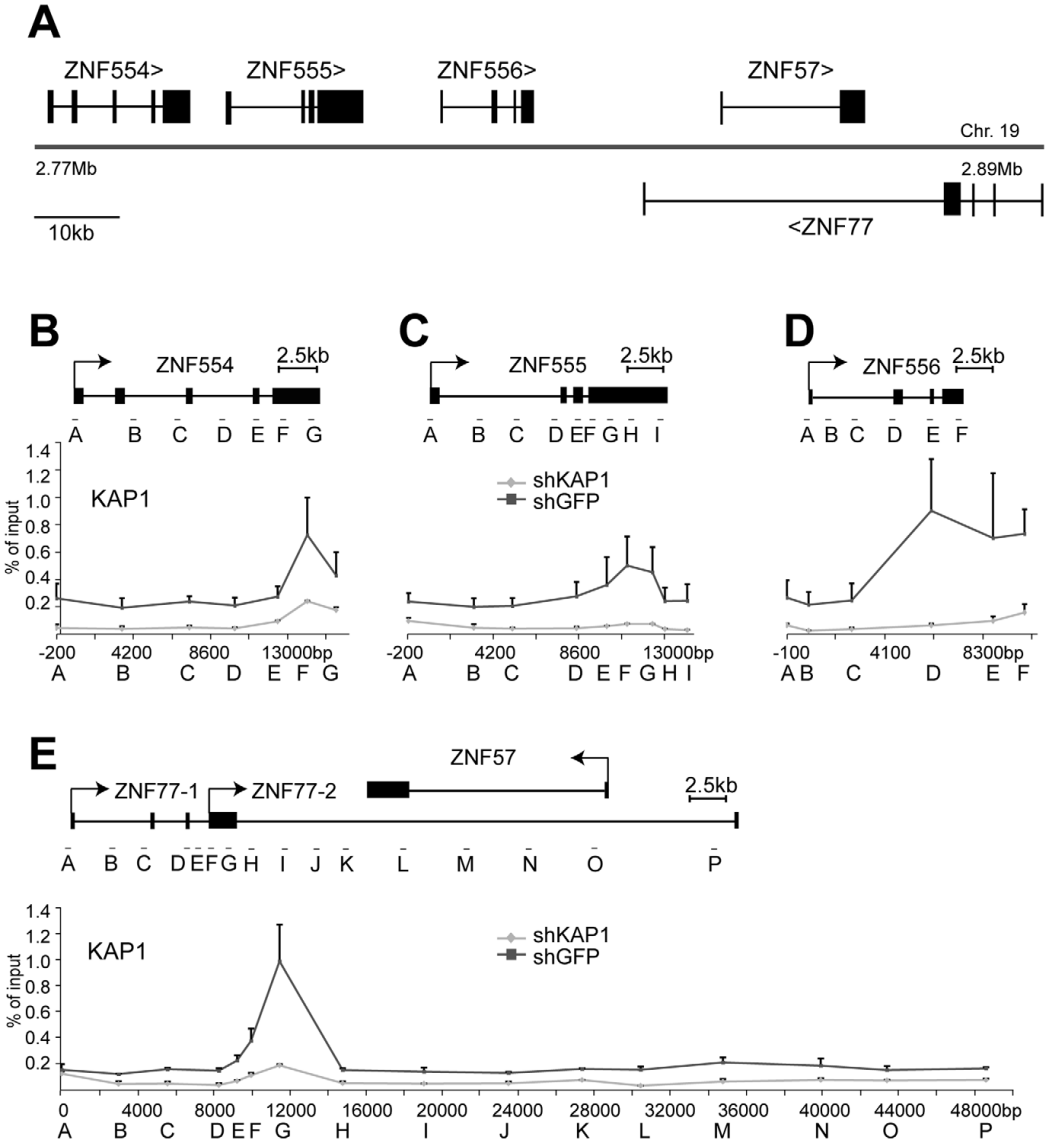
KAP1 is enriched at the 3′end of KRAB–ZFPs. (A) Depiction of the KRAB–ZFP cluster under study, located on human chromosome 19. (B–E) Direct KAP1 binding to this cluster was assessed by ChIP for the (B) ZNF554 gene, (C) ZNF555 gene, (D) ZNF556 gene, and (E) the region containing ZNF77 and the overlapping ZNF57 gene. Values are the result of duplicate experiments and are depicted with +SEM error bars.

**Figure 7:**
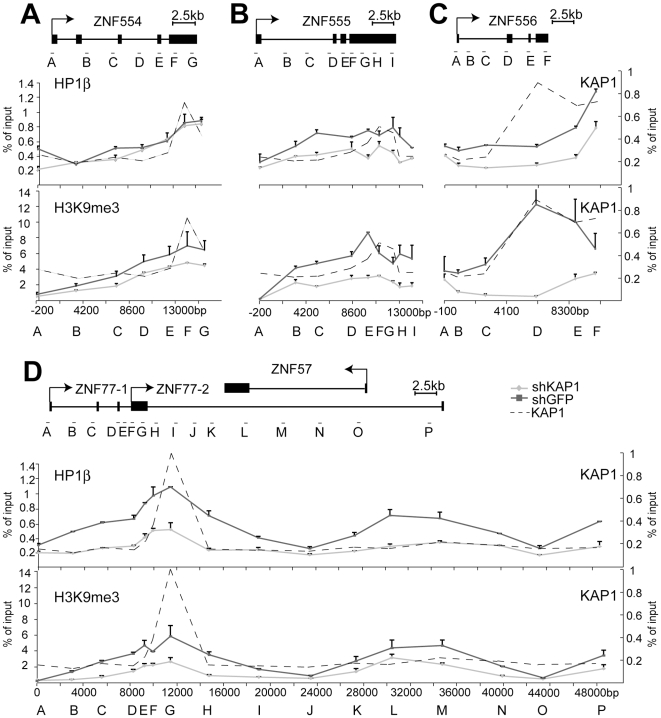
KAP1 mediates the spread of HP1β and H3K9me3 at KRAB–ZFP clusters. ChIP-mediated measurement of HP1β and H3K9me3 in control (shGFP) and KAP1 knockdown (shKAP1) HeLa cells at the (A) ZNF554 gene, (B) ZNF555 gene, (C) ZNF556 gene, and at the (D) ZNF77/ZNF57 genes. Dotted lines depict the location of KAP1 peaks determined (see Figure 6B–6E). All values are expressed as means +SEM of duplicate experiments. We ensured that comparable amounts of HP1β and H3K9me3 ChIP material were present in our shKAP1 and shGFP cell line by comparing relative enrichment levels at control loci (Figure S8).

Recent genome-wide studies have identified KAP1 and HP1β binding sites at the 3′end of KRAB-ZFP genes, suggesting long-range auto-regulatory feedback loops [38],[39]. To investigate this possibility directly, we generated a KAP1 knockdown HeLa cell line by transduction with an shRNA-expressing lentiviral vector. KAP1 mRNA and protein levels were about ten-fold lower in these cells than in their wild type control (Figure S7). We then analyzed a group of five KRAB-ZFPs located on human chromosome 19, where most members of the family reside in closely packed clusters (Figure 5, Figure 6, and Figure 7). In KAP1 knockdown cells, four out of five analyzed cluster-specific transcripts, namely ZNF554, ZNF555, ZNF556 and ZNF57, showed slight upregulation, which reached significance in the latter three genes, when compared to a cell line expressing an shRNA directed against GFP (Figure 5A). This is consistent with a de-repression of these transcripts following KAP1 depletion. The ZNF77 gene is predicted to produce two partly overlapping transcripts, ZNF77-1 and ZNF77-2. However, only ZNF77-1 was reliably detected in our cells and its levels were identical in KAP1 knockdown and control cells (Figure 5A and data not shown). We further verified that KAP1 depletion did not lead to a global decrease in heterochromatin protein expression, since four out of five analyzed genes displayed similar RNA levels in cells expressing either shKAP1 or shGFP (Figure 5B).

KAP1-specific ChIP identified KAP1 binding sites at the 3′end of ZNF554, ZNF555 and ZNF556, which are located more than 10 kb from the corresponding promoters, as well as within the region of ZNF77-1/2 overlap (Figure 6B–6E). We then examined the presence of HP1β and H3K9me3 throughout the five genes present in the cluster. In ZNF554, ZNF555, ZNF556 and ZNF77, we found peaks of HP1β and H3K9me3 enrichment at the KAP1 binding site, the intensity of which progressively decreased towards the promoter (Figure 7A–7D). We also observed elevated levels of the two silencing marks in the middle of ZNF57, for which no KAP1 association had been detected, possibly because it was not covered by our qPCR primer sets (Figure 7D). When KAP1 was targeted by shRNA, levels of HP1β and H3K9me3 decreased, consistent with KAP1 mediating the spread of silent chromatin at the affected gene bodies (Figure 7A–7D). Therefore, we conclude that KAP1 represses KRAB-ZFP gene clusters by mediating 3′ to 5′ spreading of HP1β and H3K9me3 from endogenous binding sites located at the distal end of these genes. This closely resembles results obtained with the ectopic TrapSil vectors, demonstrating the biological significance of KRAB/KAP1 in long-range repression through the propagation of heterochromatin-associated modifications.

## Discussion

In the present work, we demonstrate that KRAB-ZFPs and their co-repressor KAP1 can induce long-range transcriptional repression through heterochromatin spreading. For this, we studied lentivirally-trapped promoters silenced by an ectopic KRAB-containing repressor, as well as the KAP1-mediated modulation of the chromatin landscape at an endogenous KRAB-ZFP gene cluster. We could document the propagation of HP1β and the silencing mark H3K9me3 from repressor binding sites to targeted promoters, and showed that gene silencing results from a block in transcriptional initiation. Furthermore, we could demonstrate that this silencing process is KAP1-dependent and facilitated by HP1.

Our results demonstrate that KRAB/KAP1 have the ability to repress promoters at far greater distances than suspected based on earlier publications. Indeed, it was previously found that recruitment of a regulatable KRAB-based repressor to an array of integrated reporter genes led to transcriptional repression of the promoter adjacent to the repressor binding site, but did not affect a transcriptional unit located 2–3 kb away [37]. Here, we frequently observed repression of promoters located several tens of kilobases away from the tTRKRAB binding site, although this process was most efficient for promoters located within 15 kb or less. These results are consistent with a recent KAP1 ChIP-chip analysis, which documented the association of this co-repressor with promoters and the 3′end of KRAB-ZFP genes, and corroborate data indicating that the KRAB-ZFP ZNF263 is often found within the body of genes [38],[49]. The novel approach used here, which allows targeting of chromatin-modifying proteins to hundreds of active genes at once, provides a functional correlation to these mapping studies.

### Heterochromatin domains at KRAB–ZFP genes

The large family of KRAB-ZFP genes is the result of a recent evolutionary phenomenon, with iterative gene duplication events that have led to large sequence homologies between its different members. Therefore, it was proposed that the large heterochromatin domains containing HP1β and SUV39h1 at KRAB-ZFP genes act as a protection from deleterious recombination events, that such a homology would enhance [39]. We show here that KAP1, very likely in cooperation with specific KRAB-ZFPs, binds to the 3′end of KRAB-ZFP genes and mediates the spread of heterochromatin through their gene bodies, thus providing a molecular mechanism for the formation of these heterochromatic domains at KRAB-ZFP gene clusters. Furthermore, by examining a specific KRAB-ZFP gene cluster, we documented the KAP1-mediated control of its transcriptional units, by demonstrating increased expression of KRAB-ZFP genes upon KAP1 depletion. Importantly, the absence of silent chromatin marks at the promoters of these KRAB-ZFP genes, as well as the range of the transcriptional modulation observed, may be consistent with auto-regulatory feedback loops between different KRAB-ZFPs. We further propose that heterochromatic modifications concentrated in gene bodies of KRAB-ZFPs rather than in their promoter, ensure protection from recombination events, while preserving the transcriptional potential of KRAB-ZFP genes. Further analysis of specific KRAB-ZFPs and their binding profiles will provide more functional insight into this type of regulation.

### Mechanism of KRAB/KAP1–induced heterochromatin spreading

Spreading is considered to be an intrinsic property of heterochromatin [50]. For mammals it has been proposed that initial heterochromatin assembly at the centromere is followed by a spreading process involving HP1 and H3K9me3 in a self-perpetuating loop. It remains unclear what brings about the initial H3K9me3 that nucleates heterochromatin formation. In addition, in mammalian cells spreading of heterochromatin relies on HP1 in cooperation with additional factors such as SUV39H, since neither H3K9me2 nor H3K9me3 alone is sufficient to recruit HP1 to chromatin [51]. By analogy, KRAB/KAP1-mediated silencing may depend on similar interactions between KAP1, HP1 and H3K9me3, since long-range repression is abrogated when KAP1-HP1 binding is lost, and effective silencing is only seen if levels of H3K9me3 increase at the affected promoters themselves. In agreement with this model, recent studies on the MEST promoter, a primary KAP1 target, showed that repression is rapidly lost upon disruption of the interaction between KAP1 and HP1, and is accompanied by a decrease in H3K9me3 [52].

Remarkably, we documented a decreasing gradient of HP1β and H3K9me3 from the KAP1 docking site to the promoter in both ectopically KRAB-repressed genes and at an endogenous KRAB-ZFP gene cluster. Although it is difficult to compare the binding curves of these different factors due to the non-linear binding kinetics of the antibodies used for their capture, this may suggest that KAP1 nucleates the spread of heterochromatin. Notably, KAP1 is enriched along the bodies of all KRAB-ZFP genes (Figure 6B–6E), consistent with a model of low levels of KAP1 spreading away from KAP1 primary binding sites. It has been proposed that the propagation of heterochromatin could occur by diffusion of an enzymatically-competent macromolecular complex from a source site to nearby lower affinity sites [53]. Our data suggest that KAP1, HP1 and specific histone methyltransferases may form such a complex to mediate the spread of silent chromatin.

### Consequences of KRAB/KAP1–induced heterochromatin spreading

Our analyses of TrapSil-targeted genes and of an endogenous KRAB-ZFP gene cluster indicate not only that in both settings silent chromatin marks spread from sites of KAP1 recruitment, but also reveal that the transcriptional consequences of this event vary. Our finding that KAP1 depletion upregulated several transcripts within a KRAB-ZFP cluster, even in the absence of promoter silencing marks suggests that expression was modulated primarily by chromatin changes over the transcribed region of these genes. Therefore, our results imply broad functions for KRAB/KAP1-mediated long-range repression, encompassing not only transcriptional promoter silencing but also transcriptional dampening, as has previously been described for HP1 binding within the coding regions of genes [54],[55].

Our analysis of trapped cellular genes exposed to the tTRKRAB ectopic repressor revealed outcomes largely dictated by whether or not silencing marks reached their promoters. It is likely that the cumulative effect of increased H3K9me3 and decreased H3Ac interferes with binding of transcriptional activator proteins and the ultimate recruitment of RNA Pol II. This hypothesis is not only consistent with our finding of less hypophosphorylated RNA Pol II at trapped and silenced promoters, but was also recently put forward to explain how histone modifications regulate the expression of the L-type pyruvate kinase gene [56].

Analysis of trapped populations with different silencing phenotypes showed that the distance between sites of KRAB/KAP1 recruitment and trapped promoters had a strong influence on KRAB-mediated silencing, but was not the sole factor at play. Indeed, some promoters located at great distances from the tTRKRAB-binding site were efficiently shut off, while others situated close-by escaped this process. Several possibilities come to mind to explain this variation. First, distinct subgroups of promoters may exhibit intrinsically different susceptibility to KRAB/KAP1-mediated silencing. Second, counteracting influences may be exerted at some promoters, for instance downregulation of H3K9me3 levels by histone demethylases, as exemplified by the proposed action of JMJD1A and JMJD2C on pluripotency-associated genes in mouse ES cells [57],[58]. Third, an active nucleosome remodeling mechanism may protect a promoter from heterochromatization. This is consistent with our observation that the irrepressible trapped promoter of the casein kinase 1d locus exhibited lower levels of histone 3 upon tTRKRAB recruitment (Figure 2C). By analogy, the induction of the mammalian interferon-β promoter through external cues is also accompanied by nucleosome remodeling [59].

More generally, there may be genomic loci that exhibit chromatin environments permissive or restrictive to KRAB/KAP1-mediated long-range repression. Examples of both situations in this study include the long-range spreading of heterochromatin detected over a 50 kb long region harboring several KRAB-ZFP genes and the impaired spreading observed in the vicinity of the HNRNPA2B1-CBX3 ubiquitously-acting chromatin opening element (A2UCOE) [60]. In the first of these two cases the site of tTRKRAB recruitment at the 3′end of the ZNF77-1 gene coincided with an endogenous KAP1 binding site, so that the impact of tTRKRAB and KAP1 recruitment to this locus could be directly compared (Figure 6E and Figure S5). Their effect on promoters seemed to differ, as low basal levels of H3K9me3 at the ZNF77-1 and ZNF57 promoters dramatically increased upon tTRKRAB recruitment, which correlated with transcriptional repression. This result is consistent with our model that elevated H3K9me3 at active promoters leads to efficient transcriptional repression by KRAB/KAP1. If levels of this histone modification, however, are already low, then depletion of KAP1 will not affect the promoter as strongly. In contrast, we found that increases of both H3K9me3 and HP1β along the gene body were dependent on both KAP1 and tTRKRAB, consistent with the idea that both factors mediate the spread of heterochromatin from an initial binding site. We further speculate that specific regions, such as KRAB-ZFP gene clusters, are primed for KRAB/KAP1-mediated long-range repression, where this mechanism contributes to heterochromatin structure and long-range transcriptional repression.

In a second example we made use of trapped genes containing several TrapSil integrations with distinct silencing phenotypes (Figure S3). One of these genes is HNRNPA2B1, the promoter of which was shown to be part of a UCOE exhibiting a methylation-free CpG island (Figure S3E). This island encompasses the two divergently transcribing promoters of the housekeeping genes HNRNPA2B1 and CBX3 and allows for stable transgene expression in centromeric heterochromatin [60]. Although in our study the integrant closest to the promoter exhibited a repressible phenotype, the first irrepressible integrant was located at a distance of 3.5 kb, which is considerably lower than the observed average of 15 kb. Moreover, this gene contained a very high ratio of irrepressible/repressible integrants. It was recently shown that the DNA CpG-methylation free region spans 3 kb into the HNRNPA2B1 gene body, probably allowing for very efficient transcriptional initiation [61]. Therefore, it is conceivable that the high frequency of irrepressible clones found here is linked to the specific architecture of this promoter. We are currently trying to establish more detailed correlations between the effect of tTRKRAB-mediated silencing on genes and their individual chromatin environment.

In some cases, long-range acting elements such as enhancers might be the primary targets of KRAB/KAP1-induced chromatin modifications, with a secondary impact on promoters. More generally, specific cis-acting sequences or chromatin features located between KAP1 recruitment sites and transcriptional start sites may alter the spread of heterochromatin, either positively or negatively. Conversely, while the hereby observed long-range spreading of silencing heterochromatin suggests that some clusters of genes might be co-regulated through common KRAB-ZFP binding sites, it also implies the existence of limiting mechanisms to avoid that it systematically be the case.

### Perspectives

In conclusion, our results reveal that KRAB-ZFPs, which constitute the largest family of transcription factors encoded by the human genome, mediate long-range repression through the spread of heterochromatin in a process influenced by the genomic context. Therefore, it is tempting to speculate that the long-range epigenetic silencing (LRES) of large chromosomal regions observed in cancer cells [62] may be due to aberrant spreading of heterochromatin stemming from the dysregulation of KRAB/KAP1-mediated epigenetic regulation. Indeed, the expression of KAP1 has been found to be altered in several types of malignancies, for instance lung cancer [63],[64]. Furthermore, since tTRKRAB recruitment results in long-range repression at permissive endogenous loci, its targeting to specific chromosomal regions, through the site-specific introduction of *TetO* sequences, could be a useful tool to elucidate functions of interesting regulatory landscapes. Finally, the controlled recruitment of KRAB-containing repressors should facilitate studies on the kinetics of heterochromatin spreading in different chromosomal environments, at distinct developmental stages, and in various physiopathogical conditions, thereby shedding light on the intricacies of transcriptional repression.

## Materials and Methods

### Cell lines and culture

HeLa, 293T and HCT116 cells (from ATCC, Manassas, VA) were cultured using standard methods. Mouse embryonic fibroblasts from homozygous KAP1 floxed mice were derived as described [27]. Doxycycline (Sigma Aldrich) was used at a concentration of 1 µg/mL. GFP expression was determined by flow cytometry using the Beckton Dickinson FACScan.

### Vectors

LV- based gene trap vectors [65] were modified by introducing 7 *TetO* repeats. pLV-tTR-KRAB-Red was described [66]. LV-based plasmids expressing human wild type or mutant (R487E, V488E) KAP1 were a gift from Frank Rauscher (Wistar Institute). LV-based plasmids expressing shRNAs targeting KAP1 and GFP were purchased from Sigma and Addgene, respectively (for sequences see Table S3). LV- and MLV-based particles were produced as described [67].

### Western blot

Cells were lysed using standard procedures and subjected to SDS-PAGE. Antibodies used were mouse monoclonal anti-PCNA (Oncogene Research Products, Boston, MA) and mouse monoclonal anti-KAP1 (MAB 3662, Chemicon), followed by secondary goat anti-mouse IRDye 800CW antibody (Li-cor). Imaging and quantification of the Western blot were performed on ODYSSEY infrared imager using the machine-specific software (Li-cor).

### RNA procedures

Total RNA was isolated using an RNeasy plus kit (Qiagen).

For Northern blot 15 µg of RNA were run on a denaturing agarose gel and transferred onto a nylon membrane, which was pre-hybridized in ULTRAhyb buffer (Ambion) and after addition of a GFP-specific DIG-labelled probe (Roche, PCR DIG probe synthesis) hybridized overnight at 42°C. The membrane was then subjected to washing and detection procedures as recommended by the manufacturer (Roche, DIG Northern kit). For 5′RACE a previously described protocol was used [68]. Briefly, RNA was converted into cDNA with Super Script II Reverse Transcriptase (Invitrogen) and the trap-specific reporter primer RSP-6 or RSP-8 (for sequences see Table S3). Primary PCR reactions were performed using 2 µl of dA-tailed cDNA products and the reporter specific primers IRES(−) or RSP-6 and anchoring primers QA and QT. Nested PCR reactions were done using the reporter specific primers IRES1b or IRES(−) and anchoring primer QB. PCR products were gel purified and cloned into pCR4 by using a TOPO TA cloning kit (Invitrogen) and subsequently sequenced. Sequences were extracted from chromatograms using phred [69]. pCR4 and virus specific sequences were identified with BLAT [70] and removed prior to annotation. The location of each amplified cDNA fragment was determined with a blastn search for exact matches on the human genome (ensembl version, August 2007). Since most integrations were intronic and thus not present in the amplified cDNA, we arbitrarily assigned the proviral integration to the middle of the intron.

For quantitative Real-Time PCR (qPCR), RNA was converted into cDNA as in 5′RACE, however using random hexamer primers. For each sample a control reaction lacking RT was included. Diluted cDNA was used to assay the expression of each gene with 1x Power Sybr (Applied Biosystems), and a 400 nM concentration of each gene-specific primer. To verify specificity, each PCR was followed by a melting curve analysis. The increase in fluorescence was analysed with the SDS software, version 2.2.2 (Applied Biosystems). For all amplification plots the baseline data were set with the automatic cycle threshold function. A mean quantity was calculated from duplicate or triplicate PCRs for each sample, and a normalization factor was obtained by calculating the geometric mean of the values of the selected housekeeping genes, which was subsequently used to normalize the relative amounts of RNAs of interest [71].

### Ligation-mediated PCR (LM–PCR)

Integration sites were mapped by LM-PCR using a previously described protocol [44],[45]. Integration site determination was processed by selecting integrations site amplicons satisfying all quality controls (4/5/4 primer sequence and co-amplified viral genome fragment) and using FetchGWI [72] for alignment on the March 2006 Human Genome assembly. The gene list used for integrant mapping was downloaded from the UCSC table browser by selecting “UCSC known genes”, further including SpID and genesymbol fields. TxStart and TxEnd were downloaded to determine the transcriptional start site and 3′ end for corresponding genes.

### Chromatin immunoprecipitation (ChIP)

ChIP was performed according to the Upstate protocol with minor modifications (http://www.millipore.com/techpublications/tech1/mcproto407). About 10^7^ cells were crosslinked with 1% formaldehyde for 15 min at RT, quenched by adding glycine and rinsed with PBS. Cells were then resuspended in lysis buffer and sheared by sonication with a Branson digital sonicator (model 250) on ice four times for 20 s. 100 µl of sonicated chromatin was directly de-crosslinked and used as the total input (TI) reference in quantitative PCR analysis at a dilution of 1∶350 or 1∶700. 100 µl of sonicated chromatin was used for each ChIP reaction and was diluted in 900 µl dilution buffer and precleared with 80 µl salmon-sperm DNA- proteinA agarose beads (Upstate). Precipitating antibodies H3Ac (Upstate, 06-599), H3K9me3 (Abcam ab 8898), total H3 (Abcam ab 1791), HP1β (Abcam, ab 49938), RNA Pol II 8WG16 (Abcam, ab 817) and rabbit polyclonal KAP1 (gift from D. Schultz) were added overnight (IP). Chromatin-antibody complexes were then captured, washed and eluted with 100 mM NaHCO_3_, 1% SDS. Cross-links between DNA and proteins were reversed by addition of NaCl (for H3Ac, H3K9me3, total H3, HP1β) and incubation at 65°C. DNA was precipitated after incubation with RNase A and Proteinase K and resuspended in 50 µl H_2_O and subjected to quantitative PCR analysis at a dilution of either 1∶4 or 1∶8. Quantitative PCR was done as stated in the RT-PCR section and primer sequences are available in Table S3. Negative control reactions without antibody were run for each sample and in all cases gave negligible results. To quantify the relative enrichment of proteins or specific histone modifications at a given sequence a ratio between the relative quantities of IP and TI was calculated with the help of a standard curve and expressed as the % of input value. In the case of H3Ac, total H3 and RNA Pol II these values were further normalized to the relative enrichment value of GAPDH. In all the other cases ChIP efficiency was comparable between samples as measured at specific control loci, including p53BP2 for H3K9me3 [73] and ZNF556 for HP1β (Figure S6 and Figure S8). Fold changes were calculated as ratios of -dox/+dox enrichments, with the ratios of the respective positive controls set as 1, the controls consisted of p53BP2 for H3K9me3 and of ZNF556 for HP1β (Table S4).

## Supporting Information

Figure S1 KAP1 is recruited to the provirus of repressible and irrepressible clones. The relative enrichment (% of input) of KAP1 was quantified by ChIP analyses on the provirus of the irrepressible casein kinase 1d (CSNK1D) gene, the repressible prostaglandin E synthase 3 (PTGES3) gene and at the repressible ephrin receptor B4 (EPHB4) gene. All values are expressed as means +SEM of duplicate experiments.
(0.06 MB TIF)

Figure S2 tTRKRAB mediates silencing of retrovirally trapped cellular promoters in a selected HeLa cell population. (A) Mechanism of promoter trapping and dox-controllable silencing following TrapSil-mediated transduction of tTRKRAB-expressing cells. (B,C) tTRKRAB-expressing HeLa cells infected with LV- or MLV-derived TrapSil vectors were selected in puromycin before dox withdrawal and analysis by (B) FACS (GFP positive cells are in upper left quadrant) and (C) Northern blot (using a GFP-specific probe). The lower panels show a longer exposure of the blot and the 28S rRNA loading control.
(2.17 MB TIF)

Figure S3 Trapped genes carrying multiple TrapSil integrants of different silencing phenotypes. (A–F) Graphic depiction of six trapped genes carrying multiple LV-based TrapSil integrants with different silencing phenotypes: (A) calnexin precursor (CANX), (B) nucleophosmin 1 (NPM1), (C) sodium-coupled neutral amino acid transporter A2 (SLC38A2), (D) Y-box binding protein 1 (YBX1), (E) heterogeneous nuclear ribonucleoproteins A2/B1 (HNRNPA2B1), and (F) HNRNP C1/C2 (HNRNPC). The proviruses exhibiting a repressible phenotype (>90% silencing) are depicted as black triangles above the baseline, whereas the irrepressible counterparts (<10% silencing) are depicted as light grey triangles below the baseline.
(0.27 MB TIF)

Figure S4 HP1β and H3K9me3 spread from the KRAB-binding site to the promoter. ChIP analyses quantifying the relative enrichment (% of input) of both HP1β and H3K9me3 were performed for the repressible clone IX in the presence and absence of tTRKRAB binding. The interrogated sequence at the ephrin receptor B4 (EPHB4) locus spanned from the proviral tTRKRAB binding sites (light grey circles) to the trapped promoter. qPCR amplicons are depicted as letters and are not drawn to scale. All values are expressed as means +SEM of triplicate experiments. Fold changes were calculated as ratios of -dox/+dox enrichments, with the ratios of the respective positive controls set as 1. The controls consisted of p53BP2 for H3K9me3 and of ZNF556 for HP1β (Table S4).
(0.12 MB TIF)

Figure S5 HP1β and H3K9me3 spread along the 50 kb-spanning ZNF77/57 locus upon tTRKRAB binding. ChIP analyses quantifying the relative enrichment (% of input) of both HP1β and H3K9me3 were performed for the repressible clone XI in the presence and absence of dox. The ZNF77-1 promoter drives the expression of the integrated TrapSil provirus (depicted as light grey circles). The interrogated sequence spanned the whole ZNF77/57 locus and the respective qPCR amplicons are depicted as letters and are not drawn to scale. All values are expressed as means +SEM of triplicate experiments. Fold changes were calculated as ratios of -dox/+dox enrichments, with the ratios of the respective positive controls set as 1. The controls consisted of p53BP2 for H3K9me3 and of ZNF556 for HP1β (Table S4).
(0.30 MB TIF)

Figure S6 ChIP analyses of different TrapSil clones at control loci. (A–D) We ensured that there were similar amounts of ChIP material in the +dox compared to the -dox samples for each TrapSil clone by analyzing HP1β or H3K9me3 relative enrichment levels at control loci. These control loci are: p53BP2, ZNF554, ZNF555, ZNF556, ZNF77-1, and ZNF77-2 and were probed in the TrapSil clones (A) XVI, (B) I, (C) XI, and (D) IX.
(0.37 MB TIF)

Figure S7 Quantification of the KAP1 knockdown efficiency in a stable HeLa cell line. HeLa cells were stably transduced with lentiviruses expressing shRNA targeting either KAP1 or GFP. The levels of knockdown were quantified by using (A) qPCR measurements normalized to EEF1α and (B) western blot analyses for KAP1 levels with PCNA as a loading control. The qPCR values are expressed as means +SEM of triplicate experiments.
(0.12 MB TIF)

Figure S8 ChIP analyses of knockdown HeLa cell lines at control loci. We ensured that there were similar amounts of ChIP material in the shKAP1, compared to the shGFP cell lines, by analyzing HP1β or H3K9me3 relative enrichment levels at the control gene p53BP2 and at the human satellite 2 repeats (Sat2).
(0.08 MB TIF)

Table S1 Characterization and integration site mapping of LV-based TrapSil HeLa and MEF clones.
(0.08 MB DOC)

Table S2 Integration site mapping of the repressible and the irrepressible LV TrapSil populations. After proviral integration site mapping, the position of TrapSil integrants was determined relative to the trapped promoter for both the repressible and the irrepressible populations. More specifically, we computed two gene lists (LV repressible and LV irrepressible), which included all the trapped promoters with a single annotated transcriptional start site (TSS), which were further used to build Figure 1C. Each of these gene lists are represented by a table containing gene name (column 1), gene length (column 2), and integrant coordinate relative to the TSS of the gene (column 3). The gene name column can contain more than one identifier due to UCSC known gene annotation, which merges different gene annotations.
(0.06 MB XLS)

Table S3 List of primers used in this study.
(0.05 MB XLS)

Table S4 Fold change values calculated for different TrapSil clones by comparing ChIP enrichments in the presence or absence of tTRKRAB binding.
(0.04 MB XLS)
