# Supplementary figures and images for: KRAB–Zinc Finger Proteins and KAP1 Can Mediate Long-Range Transcriptional Repression through Heterochromatin Spreading
Source: PLoS Genet. 2010 Mar 5;6(3):e1000869. doi: 10.1371/journal.pgen.1000869 (PMC2832679; doi:10.1371/journal.pgen.1000869)

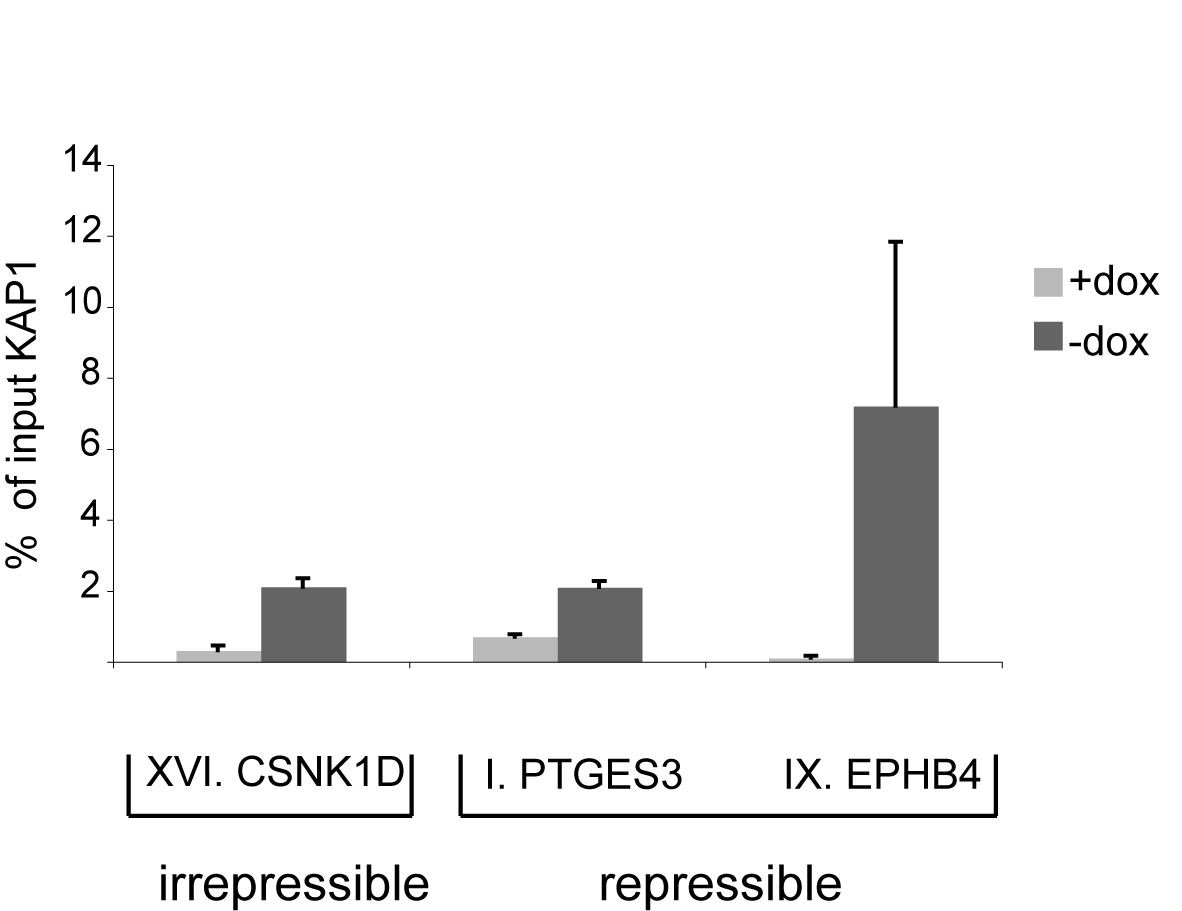

Supplement: Figure S1 — KAP1 is recruited to the provirus of repressible and irrepressible clones. The relative enrichment (% of input) of KAP1 was quantified by ChIP analyses on the provirus of the irrepressible casein kinase 1d (CSNK1D) gene, the repressible prostaglandin E synthase 3 (PTGES3) gene and at the repressible ephrin receptor B4 (EPHB4) gene. All values are expressed as means +SEM of duplicate experiments. (0.06 MB TIF) [file pgen.1000869.s001.tif]

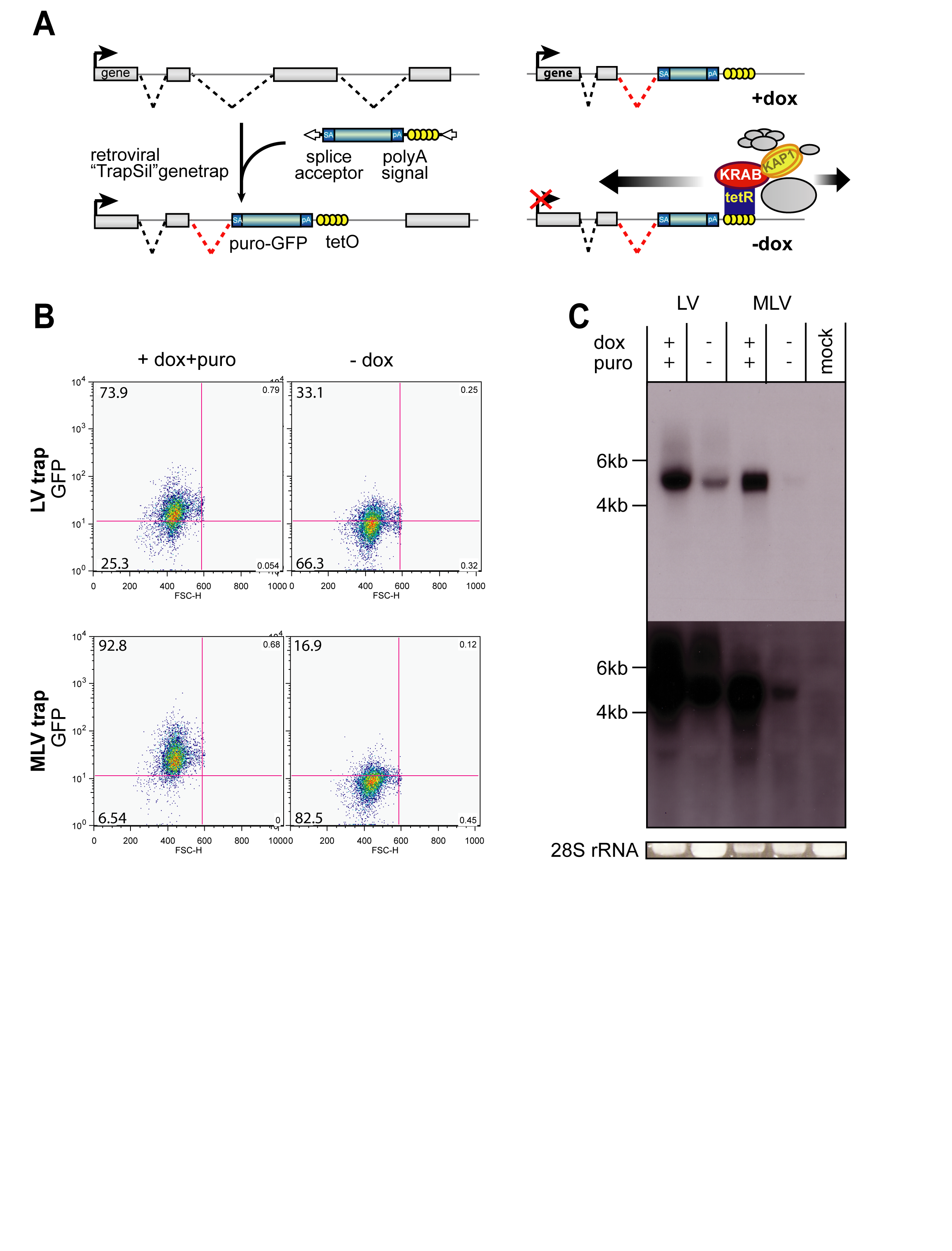

Supplement: Figure S2 — tTRKRAB mediates silencing of retrovirally trapped cellular promoters in a selected HeLa cell population. (A) Mechanism of promoter trapping and dox-controllable silencing following TrapSil-mediated transduction of tTRKRAB-expressing cells. (B,C) tTRKRAB-expressing HeLa cells infected with LV- or MLV-derived TrapSil vectors were selected in puromycin before dox withdrawal and analysis by (B) FACS (GFP positive cells are in upper left quadrant) and (C) Northern blot (using a GFP-specific probe). The lower panels show a longer exposure of the blot and the 28S rRNA loading control. (2.17 MB TIF) [file pgen.1000869.s002.tif]

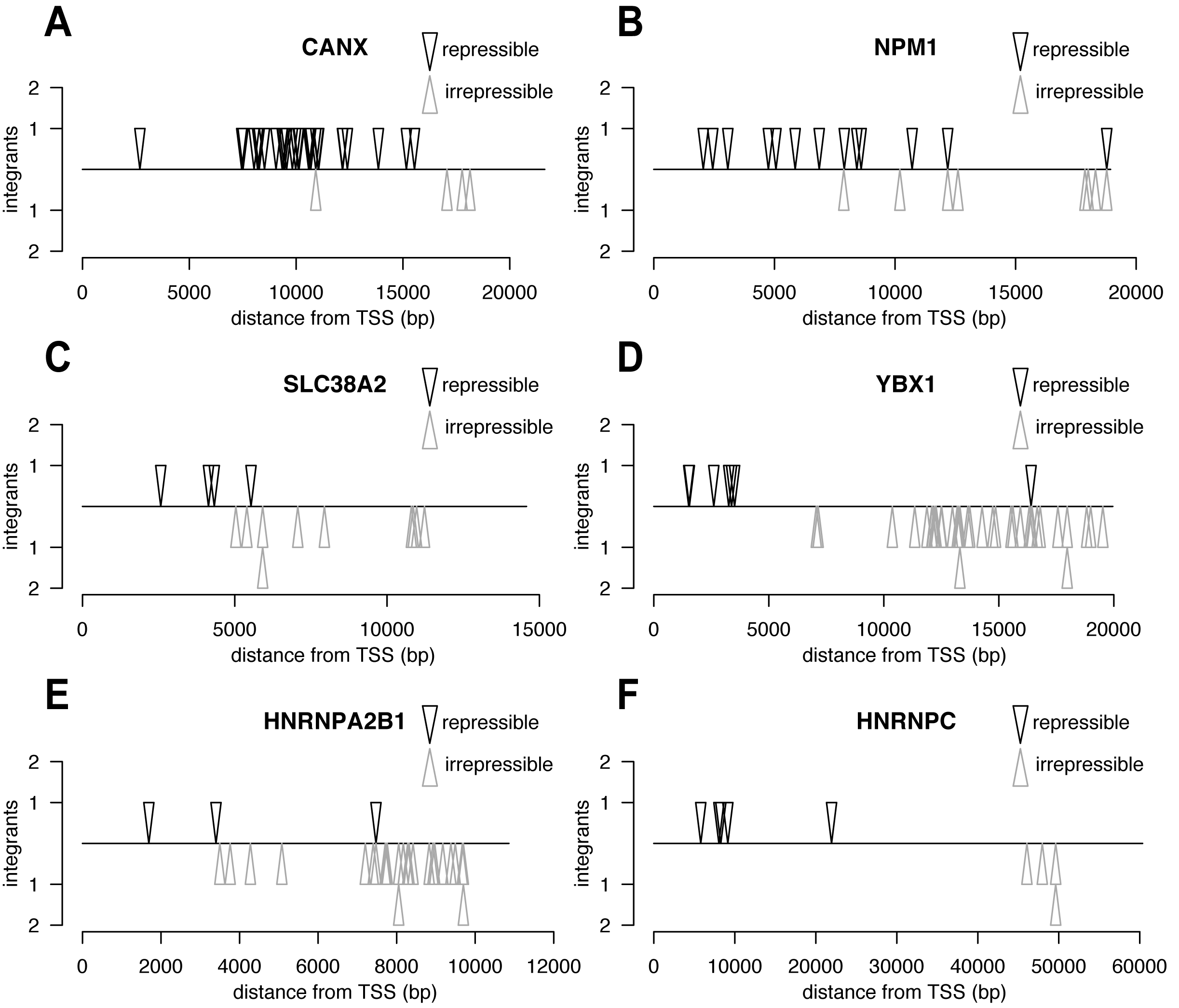

Supplement: Figure S3 — Trapped genes carrying multiple TrapSil integrants of different silencing phenotypes. (A–F) Graphic depiction of six trapped genes carrying multiple LV-based TrapSil integrants with different silencing phenotypes: (A) calnexin precursor (CANX), (B) nucleophosmin 1 (NPM1), (C) sodium-coupled neutral amino acid transporter A2 (SLC38A2), (D) Y-box binding protein 1 (YBX1), (E) heterogeneous nuclear ribonucleoproteins A2/B1 (HNRNPA2B1), and (F) HNRNP C1/C2 (HNRNPC). The proviruses exhibiting a repressible phenotype (>90% silencing) are depicted as black triangles above the baseline, whereas the irrepressible counterparts (<10% silencing) are depicted as light grey triangles below the baseline. (0.27 MB TIF) [file pgen.1000869.s003.tif]

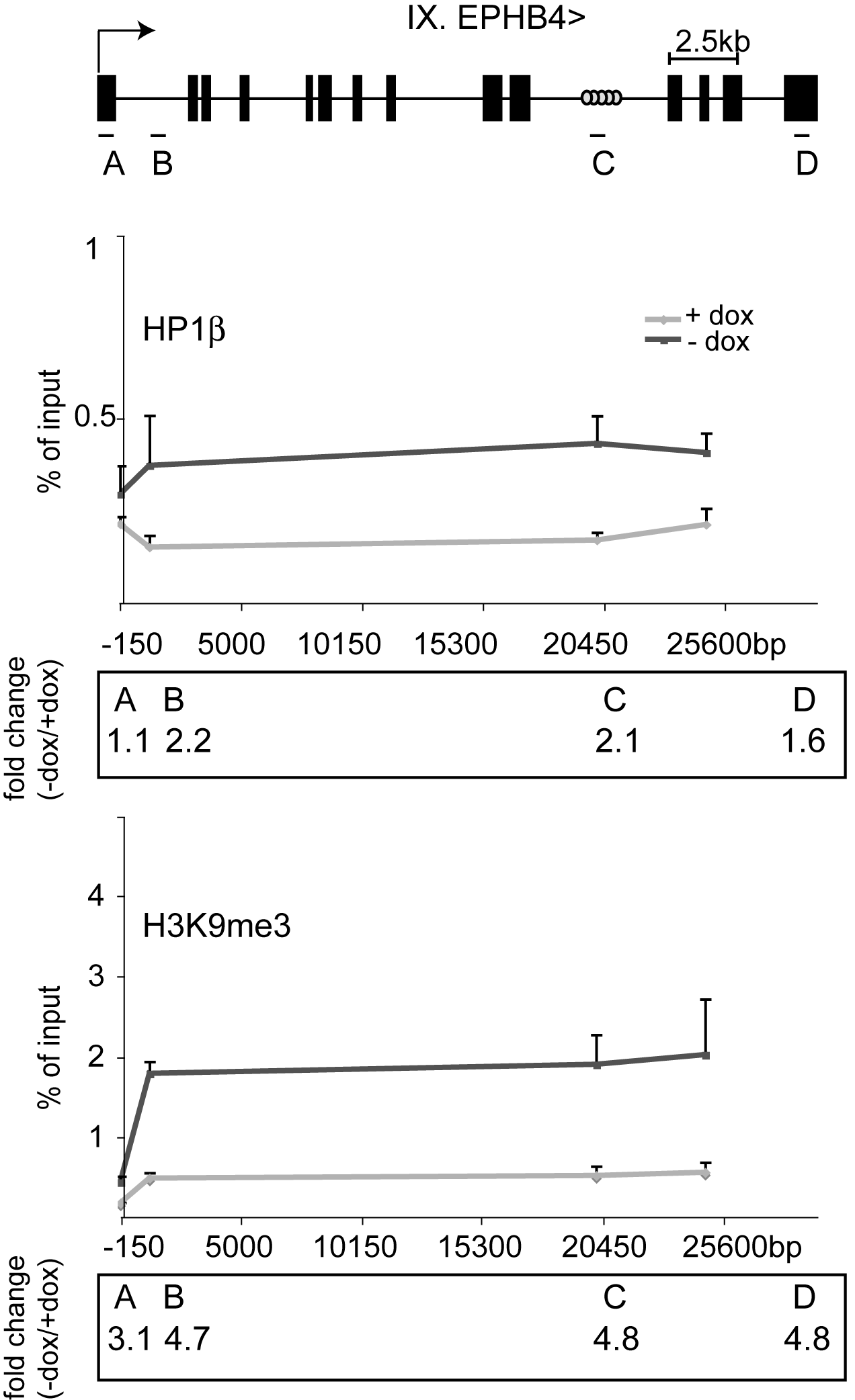

Supplement: Figure S4 — HP1β and H3K9me3 spread from the KRAB-binding site to the promoter. ChIP analyses quantifying the relative enrichment (% of input) of both HP1β and H3K9me3 were performed for the repressible clone IX in the presence and absence of tTRKRAB binding. The interrogated sequence at the ephrin receptor B4 (EPHB4) locus spanned from the proviral tTRKRAB binding sites (light grey circles) to the trapped promoter. qPCR amplicons are depicted as letters and are not drawn to scale. All values are expressed as means +SEM of triplicate experiments. Fold changes were calculated as ratios of -dox/+dox enrichments, with the ratios of the respective positive controls set as 1. The controls consisted of p53BP2 for H3K9me3 and of ZNF556 for HP1β (Table S4). (0.12 MB TIF) [file pgen.1000869.s004.tif]

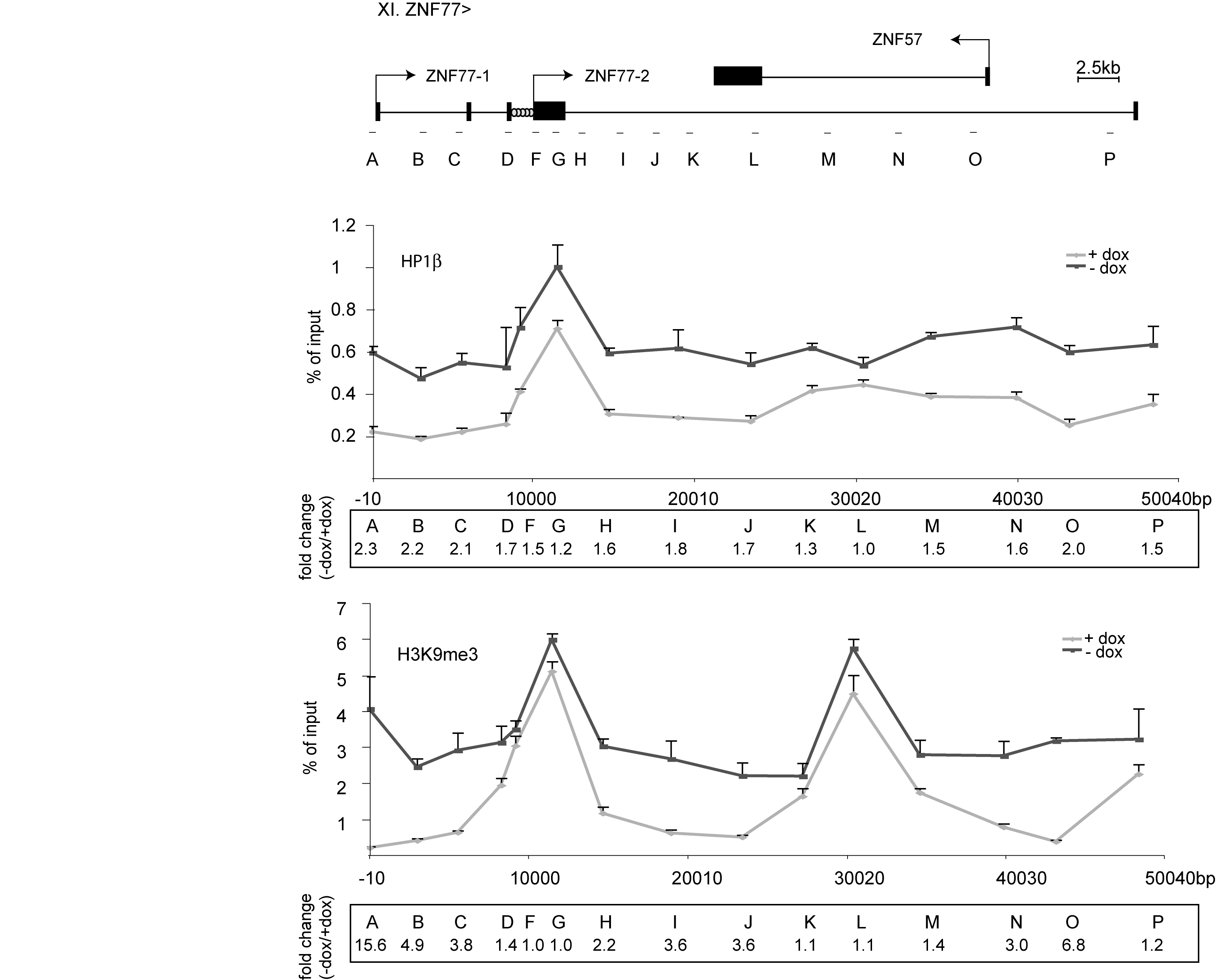

Supplement: Figure S5 — HP1β and H3K9me3 spread along the 50 kb-spanning ZNF77/57 locus upon tTRKRAB binding. ChIP analyses quantifying the relative enrichment (% of input) of both HP1β and H3K9me3 were performed for the repressible clone XI in the presence and absence of dox. The ZNF77-1 promoter drives the expression of the integrated TrapSil provirus (depicted as light grey circles). The interrogated sequence spanned the whole ZNF77/57 locus and the respective qPCR amplicons are depicted as letters and are not drawn to scale. All values are expressed as means +SEM of triplicate experiments. Fold changes were calculated as ratios of -dox/+dox enrichments, with the ratios of the respective positive controls set as 1. The controls consisted of p53BP2 for H3K9me3 and of ZNF556 for HP1β (Table S4). (0.30 MB TIF) [file pgen.1000869.s005.tif]

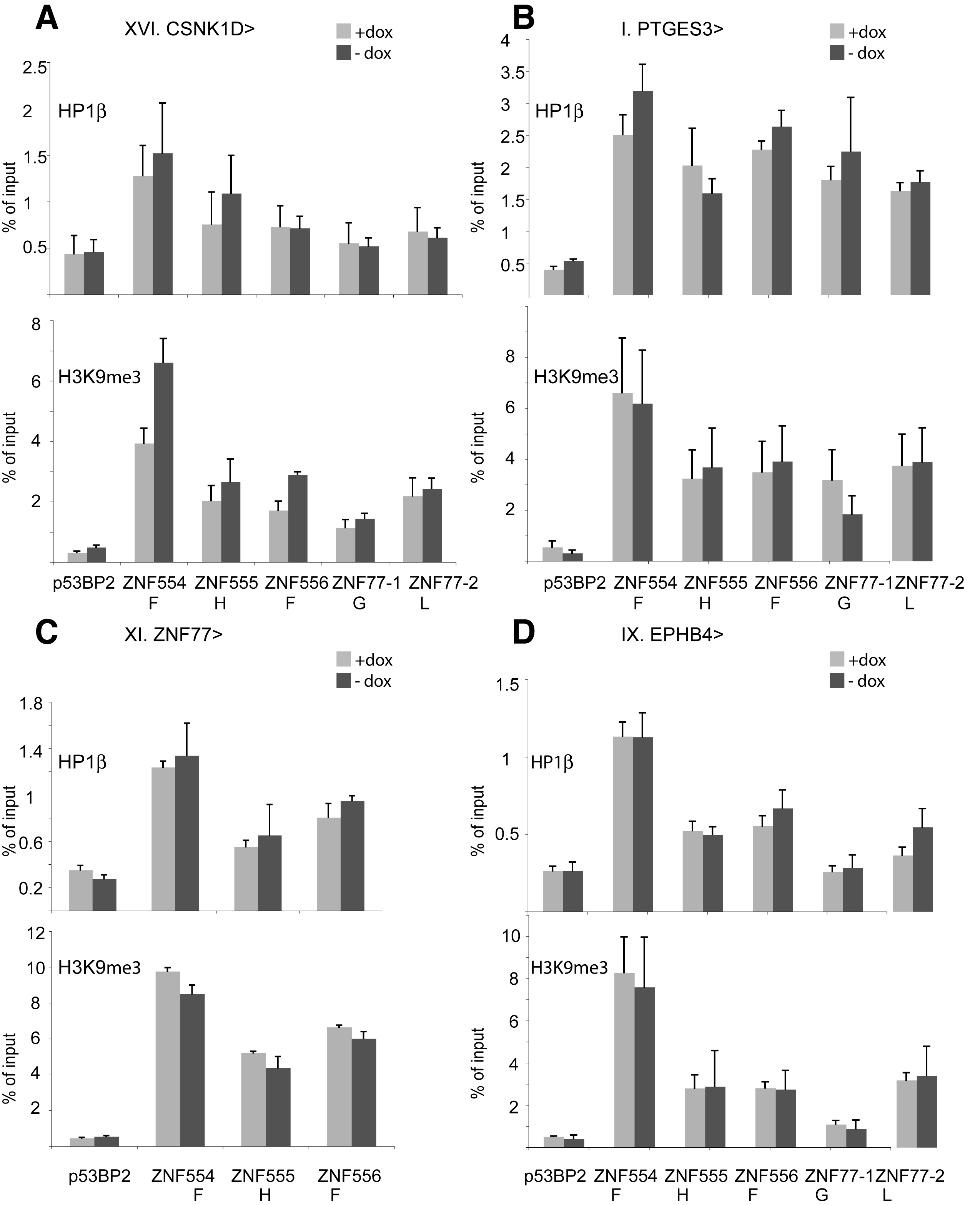

Supplement: Figure S6 — ChIP analyses of different TrapSil clones at control loci. (A–D) We ensured that there were similar amounts of ChIP material in the +dox compared to the -dox samples for each TrapSil clone by analyzing HP1β or H3K9me3 relative enrichment levels at control loci. These control loci are: p53BP2, ZNF554, ZNF555, ZNF556, ZNF77-1, and ZNF77-2 and were probed in the TrapSil clones (A) XVI, (B) I, (C) XI, and (D) IX. (0.37 MB TIF) [file pgen.1000869.s006.tif]

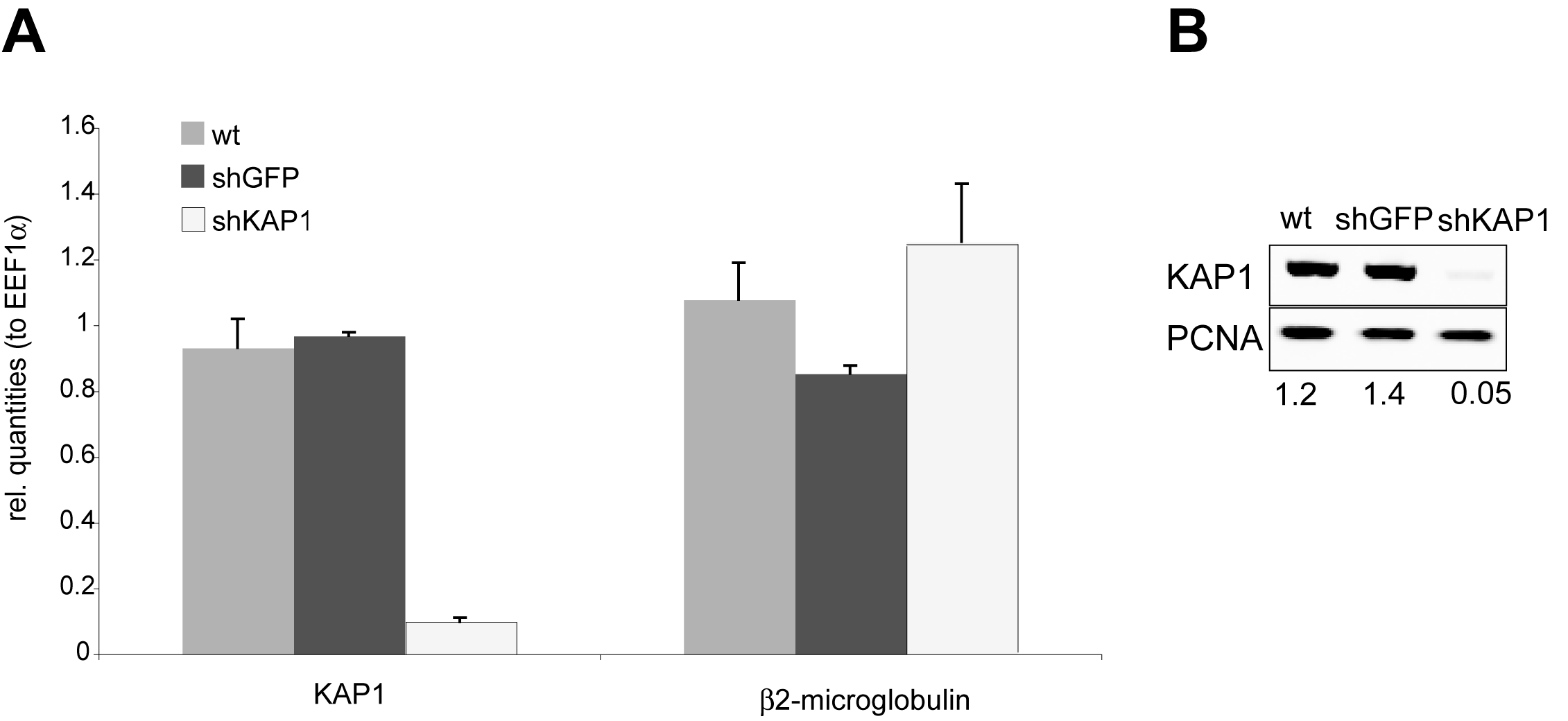

Supplement: Figure S7 — Quantification of the KAP1 knockdown efficiency in a stable HeLa cell line. HeLa cells were stably transduced with lentiviruses expressing shRNA targeting either KAP1 or GFP. The levels of knockdown were quantified by using (A) qPCR measurements normalized to EEF1α and (B) western blot analyses for KAP1 levels with PCNA as a loading control. The qPCR values are expressed as means +SEM of triplicate experiments. (0.12 MB TIF) [file pgen.1000869.s007.tif]

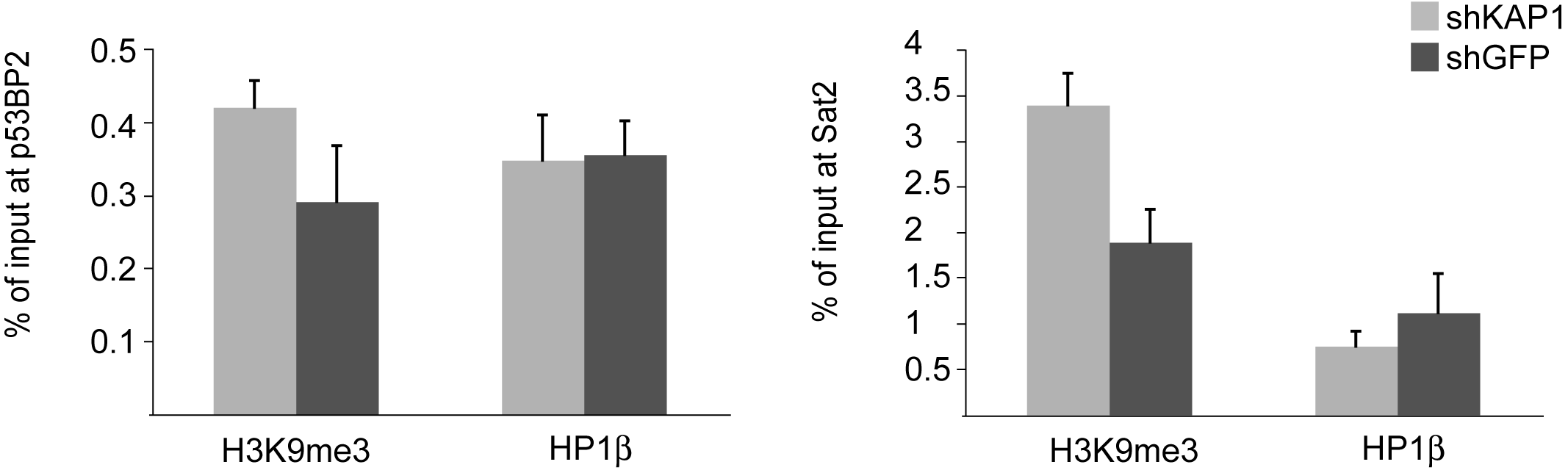

Supplement: Figure S8 — ChIP analyses of knockdown HeLa cell lines at control loci. We ensured that there were similar amounts of ChIP material in the shKAP1, compared to the shGFP cell lines, by analyzing HP1β or H3K9me3 relative enrichment levels at the control gene p53BP2 and at the human satellite 2 repeats (Sat2). (0.08 MB TIF) [file pgen.1000869.s008.tif]
