# Supplementary material for: KRAB–Zinc Finger Proteins and KAP1 Can Mediate Long-Range Transcriptional Repression through Heterochromatin Spreading
Source: PLoS Genet. 2010 Mar 5;6(3):e1000869. doi: 10.1371/journal.pgen.1000869 (PMC2832679; doi:10.1371/journal.pgen.1000869)
Supplement: Table S1 — Characterization and integration site mapping of LV-based TrapSil HeLa and MEF clones. (0.08 MB DOC) [file pgen.1000869.s009.doc]

**Supplementary table S1:**

# LV-“TrapSil” HeLa cell clones

| clone | Silencing% | Method mapping | Gene | Distance to TSS (kb) * |
| --- | --- | --- | --- | --- |
| I (L10) | 96.30% | 5’ RACE | PTGES3 | 20 |
| II (C3DLV16) | 95.99% | LM PCR | ZNF10-201 | 12 |
| III (C3DLV10) | 94.56% | LM PCR | ST13 | 22 |
| IV (C3DLV9) | 81.54% | LM PCR | ATL3 | 25 |
| V (L25) | 78.60% | 5’ RACE | SAFB23 | 8.1 |
| VI (L11) | 79.50% | 5’ RACE | NDUFS2 | 5.5 |
| VII (L27) | 99.70% | 5’ RACE | CDCA5 | 2.5 |
| VIII (L2) | 99.60% | 5’ RACE | Hsp90AB1 | 4 |
| IX (L20) | 83.60% | 5’ RACE | EC2.7/EPHB4 | 17.4 |
| X (L7) | 44.20% | 5’ RACE | ZGPAT | 12.5 |
| XI (L12) | 99.40% | 5’ RACE | ZNF77 | 9.25 |
| XII (L15) | 53.60% | 5’ RACE | GPATCH8 | 73.5 |
| XIII (L17) | 99.30% | 5’ RACE | Nup54 | 8.2 |
| XIV (L18) | 63.30% | 5’ RACE | MTDC | 3.5 |
| XV (C3LV3) | 10.07% | LM PCR | CAPRIN1/Q14444 | 11 |
| XVI (L9) | 14.40% | 5’ RACE | KC1D | 22.5 |
| XVII (C3LV16) | 22.55% | LM PCR | FNBP4 | 5 |
| XVIII (C3LV12) | 3.39% | LM PCR | SYNCRIP | 22 |
| XIX (L13) | 38.90% | 5’ RACE | CCDC47 | 20 |
| XX (L22) | 57.80% | 5’ RACE | SNX12 | 12 |
| XXI (L23) | 33.80% | 5’ RACE | TMEM123 | 26 |
| XXII (L8) | 41.70% | 5’ RACE | LDC2/RNPS1 | 14 |
| XXIII (L24) | 52.40% | 5’ RACE | Arf1 | 7.2 |

# * The distance to the transcriptional start site is an estimate value for 5’RACE, where intronic integrations were assumed to have occurred in the middle of the intron downstream of the amplified exon. For LM PCR the distance is a precise value, since the proviral-genomic DNA junction is directly amplified.

# LV-“TrapSil” MEF KAP1-/- cell clones

| clone | silencing% | Method mapping | gene | distance to TSS (kb) * |
| --- | --- | --- | --- | --- |
| 1 | 0.1 | 5’RACE | Sestd1 | 15 |
| 1KAP1^wt^ | 62.5 | 5’RACE | Sestd1 | 15 |
| 1KAP1^R487E^, ^V488E^ | 9.6 | 5’RACE | Sestd1 | 15 |
| 2 | 0.5 | 5’RACE | Prcp-201 | 25 |
| 2KAP1^wt^ | 31.2 | 5’RACE | Prcp-201 | 25 |
| 2KAP1^R487E^, ^V488E^ | 5.8 | 5’RACE | Prcp-201 | 25 |

* The distance to the transcriptional start site is an estimate value for 5’RACE, where intronic integrations were assumed to have occurred in the middle of the intron downstream of the amplified exon. For LM PCR the distance is a precise value, since the proviral-genomic DNA junction is directly amplified.
